# The haplotype of three polymorphisms in the *SATB1* promoter region impacts survival in breast cancer patients

**DOI:** 10.3892/ol.2014.1983

**Published:** 2014-03-20

**Authors:** MARTIN HEUBNER, RAINER KIMMIG, BAHRIYE AKTAS, WINFRIED SIFFERT, ULRICH H. FREY

**Affiliations:** 1 Institute of Pharmacogenetics, University of Duisburg-Essen, Essen D-45122, Germany; 2 Department of Obstetrics and Gynaecology, University of Duisburg-Essen, Essen D-45122, Germany; 3 Department of Anesthesiology and Intensive Care Medicine, University of Duisburg-Essen, Essen D-45122, Germany

**Keywords:** *SATB1*, breast cancer, SNP, polymorphism, haplotype, promoter

## Abstract

Special AT-rich sequence binding protein 1 (SATB1) has regulatory effects on gene expression and appears to play an important role in tumor progression. The present study screened the promoter region of the *SATB1* gene for polymorphisms, evaluated the corresponding haplotypes regarding alterations in promoter activity *in vitro* and analyzed the impact of these haplotypes on the clinical course of breast cancer patients. A cohort of 241 female Caucasian breast cancer patients who had been treated was enrolled in this retrospective analysis. The median follow-up time was 93 months (range, 4–155 months). PCR products from DNA of 10 healthy, unrelated volunteers were analyzed to identify new polymorphisms within the promoter region. Genotyping was conducted using restriction fragment length polymorphism and pyrosequencing. PCR constructs with the respective alleles from the four most frequent haplotypes were cloned into the vector pGEM^®^-T Easy and then transferred into the *luc2*-containing reporter vector pGl 4.10^®^ for transfection of HEK293 cells. The pGl 4.73^®^ vector, containing *hRluc*, was used for normalizing the transfection rates. Sequencing the region -3807 to -2828 bp upstream of ATG from ten healthy blood donors, three single nucleotide polymorphisms consisting of base exchanges were identified: -3600T>C, -3363A>G and -2984C>T. The *SATB1* -3600T/-3363A/-2984C haplotype had lower promoter activity than all other constructs *in vitro* and exhibited a significant association with nodal status (P<0.05). Kaplan-Meier survival analysis revealed significantly improved overall survival for homozygous *SATB1* -3600T/-3363A/-2984C haplotype carriers compared with heterozygous carriers or the other haplotypes (P=0.033). The *SATB1* -3600T/-3363A/-2984C haplotype is associated with lower promoter activity and appears to impact upon survival in breast cancer patients.

## Introduction

Breast cancer is the most common type of malignancy in females and remains a therapeutic challenge (1). Early-stage breast cancer is normally associated with a good prognosis. However, a considerable number of patients suffer from distant metastases which are usually life-limiting. It is therefore essential to identify these high-risk patients. Going beyond clinical and histopathological staging and grading, molecular markers correlating with prognosis have become increasingly important and are decisive factors in deciding upon adjuvant therapies (2).

The special AT-rich sequence binding protein 1 (SATB1) binds to matrix attachment regions and has regulatory effects on gene expression (3–6). SATB1 binds to heterochromatin and functions by recruiting chromatin-modifying enzymes and transcription factors (6,4). SATB1 is an important factor in the development of thymocytes and T-cells (7). It has also been identified as a silencing factor contributing to the initiation of X-inactivation (8), which makes it particularly interesting in terms of X-linked tumor suppressor genes (9).

In addition, the expression of SATB1 has been found to correlate with diminished overall survival in breast cancer patients (10). SATB1 appears to play an important role in transforming the gene expression profile of tumor cells to have invasive and metastasizing properties, and knockdown of *SATB1* has been demonstrated to result in the reversion of distant metastases (10). The *BCL2* gene, which is crucial in the regulation of apoptosis, appears to be partly regulated by interactions with SATB1 (11). Direct impact of SATB1 inhibition on tumor growth in breast cancer has been observed *in vitro* (12). Thus, SATB1 may also be an attractive therapeutic target in future.

In this study, the promoter region of the *SATB1* gene was screened for polymorphisms, the corresponding haplotypes regarding alterations in promoter activity *in vitro* were evaluated, and the impact of these haplotypes on the clinical course of breast cancer patients was analyzed.

## Patients and methods

### Patients

A cohort of 241 Caucasian female breast cancer patients who had been treated for thier first diagnosis of breast cancer between 1989 and 1993 at the Department of Obstetrics and Gynaecology, University Hospital of Essen (Essen, Germany), was enrolled in this retrospective analysis, and their clinical data were documented. Overall survival data were obtained from the local municipal registry. The median follow-up time was 93 months (range, 4–155 months). Tumor stages were classified according to the TNM and World Health Organization classifications of breast tumors (13,14). The control cohort consisted of healthy, Caucasian, age-matched female voluntary blood donors. Approval for this study was obtained from the ethics committee of the Medical Faculty, University of Duisburg-Essen (Essen, Germany) and patients gave their informed consent.

### Identification of single nucleotide polymorphisms (SNPs)

PCR products from DNA of 10 healthy, unrelated volunteers were used to identify new polymorphisms within the promoter region. Using available reference sequences of human *SATB1*, primer pairs were designed to amplify overlapping PCR products of the assumed promoter region from -3807 up to -2828 bp upstream of ATG.

DNA sequencing was performed by a third party (MWG Eurofins Medigenomix, Ebersberg, Germany). Reference sequences and sequenced fragments were analyzed using DNASTAR MegAlign^®^ (DNASTAR, Inc., Madison, WI, USA) for Windows^®^.

### Genotyping

Healthy voluntary blood donors and patients were retrospectively genotyped for *SATB1* polymorphisms. DNA was extracted from whole blood or paraffinum-embedded tumor-free tissue using a QIAamp kit (Qiagen, Hilden, Germany).

For -3600T>C, PCR was performed with the forward primer 5′-AGGCGGTGGAGGTGGCTG-3′ and the reverse primer 5′-GCGGGGCTGTGAGCGTCT-3′, resulting in a 107 bp fragment (Eurofins MWG Operon, Ebersberg, Germany). Following denaturation at 95°C, 38 cycles of DNA amplification were performed using *Taq* PCR Mastermix (Eppendorf, Hamburg, Germany) at 95°C for 40 sec, 62°C for 40 sec and 72°C for 40 sec. Digestion with *Bsa*XI (New England Biolabs, Inc., Ipswich, MA, USA) at 37°C resulted in fragments of 50, 30 and 27 bp for the C-allele versus 107 bp for the TT-genotype (no digestion). Electrophoresis was performed in 2.8% agarose gels using SYBR Safe^®^ DNA gel stain (Invitrogen Life Technologies, Carlsbad, CA, USA) for visualization under ultraviolet light.

Genotyping of -3363A>G was carried out by PCR with the forward primer 5′-GGCTGTGGGGAAAAGTTTAAGGTT-3′ and the biotinylated reverse primer 5′-CCGAATAACGCGCATTGG-3′. The annealing temperature was 62°C, and the remaining PCR conditions were as described above. The 111-bp PCR products were analyzed by pyrosequencing using the sequencing primer 5′-ATATTAGTCGCGATTGTTG-3′ on the PSQ96 system, according to the manufacturer’s instructions, and results were analyzed using the PSQ96 SNP software (Biotage AB, Uppsala, Sweden).

For -2984C>T, PCR was performed with the forward primer 5′-TTTTACGATTTCCCCCCAAC-3′ and the biotinylated reverse primer 5′-TGTAAAATGTCTAACCTCAGAGAA-3′ with an annealing temperature of 67°C and accordant PCR conditions. Pyrosequencing of the 122 bp product was performed using the sequencing primer 5′-TCCCCATCGCAAACC-3′ as described above.

For each genotyping assay, the certainty of the method was confirmed by comparison with the sequence analyses of ten healthy blood donors.

### Cloning

Primers were designed to amplify the region from -3801 up to -2801 bp upstream of ATG. The corresponding PCR products were sequenced by an external service to ensure correctness. Constructs with the respective alleles from the four most frequent haplotypes were cloned into the pGEM^®^-T Easy vector (Promega Corporation, Madison, WI, USA) and then subcloned into the *luc2*-containing reporter vector, pGl 4.10^®^ (Promega), for transfection of HEK293 cells. The pGl 4.73^®^ vector (Promega), containing *hRluc*, was used for normalizing the transfection rates.

### Transfection

Human embryonic kidney cells (HEK293) were routinely maintained in Dulbecco’s modified Eagle’s medium supplemented with 10% fetal bovine serum.

For transfection, ~20,000 HEK293 cells were seeded into 96-well dishes, and transfected by Lipofectamine 2000 (Invitrogen Life Technologies). Cells were cotransfected with 150 ng firefly reporter vector pGL 4.10^®^ containing the *SATB1* promoter fragment, and 50 ng *Renilla* luciferase vector pGL 4.73^®^ to control for transfection efficiency. After 6 h, the cell culture medium was replaced by 75 ml cell culture medium without FBS. Untransfected HEK293 cells, as well as HEK293 cells transfected with an empty pGl 4.10^®^ vector, were included as means of control.

### Dual Glo™ luciferase assay

The Dual Glo™ luciferase assay system (Promega) is designed for the functional analysis of promoter or 3′UTR regions. Regulation deriving from transcription factors as well as from posttranscriptional modifications by micro-RNAs can be detected. It is based on dual transfection with two luciferase-active vectors, one containing the corresponding construct and one untransfected vector for the purpose of transfection normalization. The reagents Dual Glo^®^ and Stop&Glo^®^ (Promega) are added sequentially to measure the activity of *luc2* and *hRluc*.

Luciferase activity was measured 24 h after transfection using a 96-well luminometer (Berthold Technologies GmbH & Co. KG, Bad Wildbad, Germany). Measurement periods were 1 sec for *luc2* activity and 5 sec for *hRluc* activity. The background activity was subtracted prior to evaluation. Firefly luciferase activity was normalized for *Renilla* luciferase activity as recommended by the manufacturer (Promega).

The assessment was performed in duplicate for each construct and control. In total, six runs were carried out.

### Statistical analysis

Deviations from the Hardy-Weinberg equilibrium (HWE) were tested for using Pearson’s χ^2^ test. Haplotype analysis was conducted with Haploview 4.0^®^ for Windows (Broad Institute, Cambridge, MA, USA) as previously described (15). Genotype frequencies of patients and controls were compared using the χ^2^ test. Kaplan-Meier analysis was applied to examine the prognostic importance of the genotypes on overall survival and progression-free survival. Progression-free survival was calculated from the time of initial diagnosis to the time of diagnosed progressive disease. Overall survival was calculated from the date of first diagnosis to the date of death. Comparison of clinical and laboratory parameters between patient subgroups was performed using the Kruskal-Wallis and the Mann-Whitney U test for continuous variables and the χ^2^ test for categorical data. The log-rank test was used to compare the survival distributions of subgroups. Differences were regarded as statistically significant when P<0.05. Statistical analyses were performed using SPSS 15.0 for Windows (SPSS, Inc., Chicago, IL, USA).

## Results

### Detection of polymorphisms

Sequencing the region -3807 to -2828 bp upstream of ATG in 10 healthy blood donors identified three SNPs consisting of the base exchanges -3600T>C, -3363A>G and -2984C>T.

While -2984C>T (rs6762753) and -3600T>C (rs73040343) were already accessible in available reference sequences, -3363A>G is a novel polymorphism which was analyzed for the first time in this study.

### Distribution of genotypes and haplotypes in patients and controls

Genotyping patients and healthy controls revealed that the distribution of genotypes was compatible with the HWE for patients as well as for controls. Genotype analysis using the χ^2^ test and haplotype analysis did not reveal significant differences between patients and controls, suggesting that no genotype or haplotype is associated with an increased risk of breast cancer. Table I summarizes the genotyping results, including minor allele frequencies.

Haplotype analysis revealed linkage disequilibrium between the polymorphisms, as described in Table II.

### Functional assessment of haplotypes

**Figure 1:**
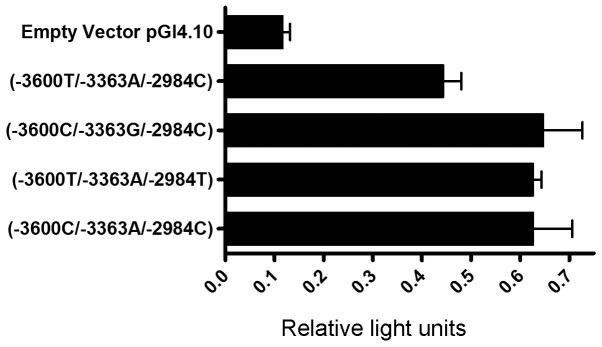
Assessment of promoter activity by Dual Glo™ luciferase assay revealed significant differences in activity between the tested DNA fragments (P=0.034, according to the Kruskal-Wallis test for continuous variables).

Analysis of luciferase activity revealed promoter activity for all haplotypes compared with the empty vector. The *SATB1* -3600T/-3363A/-2984C haplotype exhibited lower promoter activity than all other constructs (Fig. 1). Comparing the haplotypes, the observed differences in promoter activity were statistically significant (P=0.034) using the Kruskal-Wallis test for continuous variables.

### Association of genotypes and haplotypes with clinical data

Demographic and clinical data of the analyzed breast cancer patients are summarized in Table III. The median age of the patients was 56 years (range, 27–82 years) and the median follow-up time was 93 months (range, 4–155 months).

**Figure 2:**
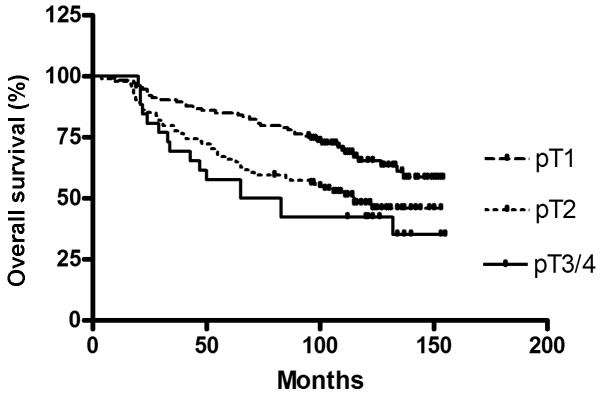
Survival of patients depending on tumor stage (P=0.0010).

**Figure 3:**
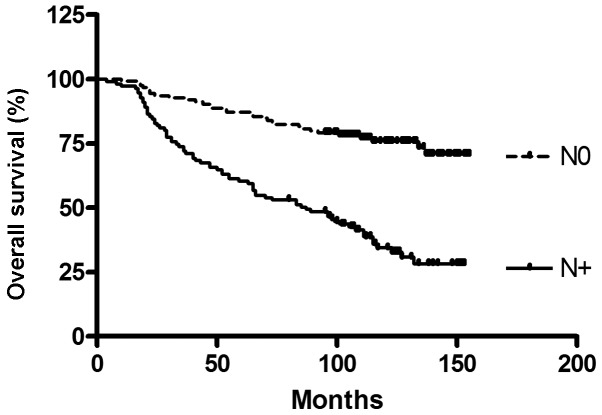
Survival of patients depending on nodal status (P<0.0001).

To confirm the established prognostic factors, of the breast cancer patient cohort, a survival analysis for tumor stage and nodal status, which are known to be predictive for survival, was conducted (Figs. 2 and 3).

The genotypes were each tested for association with clinical data, including age, tumor size, tumor stage, grading, histopathological tumor type, estrogen receptor and Her2 status, and overall survival. No significant associations regarding the solitary SNP genotypes were noted (not shown).

The functional haplotype assessment revealed significantly reduced promoter activity in the -3600T/-3363A/-2984C construct, and so further analyses were made to test associations of the *SATB1* -3600T/-3363A/-2984C haplotype with clinical data. Significantly different distributions of nodal status were identified between homozygous haplotype carriers, heterozygous haplotype carriers and non-carriers (Table III).

**Figure 4:**
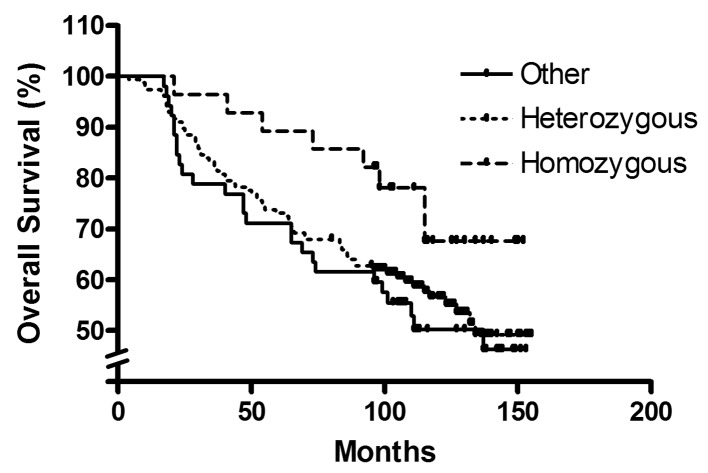
Survival of patients depending on *SATB1* -3600T/-3363A/-2984C promoter polymorphism haplotype (log-rank test, P=0.033; adjusted for nodal status).

Kaplan-Meier survival analysis revealed significantly improved overall survival for homozygous *SATB1* -3600T/-3363A/-2984C haplotype carriers compared with heterozygous carriers or other haplotypes (P=0.033, corrected for nodal status; Fig. 4).

## Discussion

SATB1 has high prognostic relevance in breast cancer patients. Therefore, it appeared probable that genetic variations resulting in altered promoter activity may influence the course of disease in these patients.

Several polymorphisms in the *SATB1* promoter region were identified, and the accordant haplotypes were found to alter the promoter activity and overall survival rates of breast cancer patients. Regarding the functional promoter assay, the clinical results fit well into what is currently known about *SATB1*. As the -3600T/-3363A/-2984C haplotype demonstrates lower activity, it would therefore lead to a diminished transcription rate. The association of this haplotype with improved prognosis is thus what would be expected from these experimental findings.

A significant correlation between the -3600T/-3363A/-2984C haplotype and the nodal status of breast cancer patients was identified, although this association was weak. However, *SATB1* may influence other prognostic parameters, as previous authors have reported associations with tumor stage and tumor grading (16).

Considering that the haplotype associated with improved prognosis is also the most common one, it could be argued that these findings are of minor interest compared with other prognostic markers. However, the majority of patients had at least heterozygous status, which in itself resulted in diminished overall survival. The consequences for the affected patients are not yet clear. However, we are currently in an era of molecular therapeutic approaches, which may focus on *SATB1* in the near future. If there were attempts at therapeutic inhibition, patients who do not carry the SNP may be the ones who would benefit the most from these efforts.

One of the limitations of the present study is the fact that that the promoter region of *SATB1* has not yet been functionally described. However, the experimental and clinical findings of the study support the hypothesis that the analyzed sequence is part of the promoter. Notably, the -3600T/-3363A/-2984C haplotype is associated with nodal status. This association may be explained, as the observed effect of the haplotype on promoter activity may alter the risk of early lymphangio-invasion in the primary tumor.

In future, *SATB1* may be of interest in terms of adjuvant, neoadjuvant or palliative chemotherapy. *In vitro* analyses suggest that *SATB1* may play a role in mechanisms of multidrug resistance, as its depletion results in enhanced drug sensitivity to cytostatic drugs (17).

*SATB1* is a relatively new prognostic parameter, and its significance in breast cancer is controversial (9,18). However, previous data suggest a role of *SATB1* in the progression and metastasis of other tumor types, including non-small-cell lung cancer, gastric cancer and melanoma (19–21).

It is of interest whether the prognostic impact of the described haplotype also applies for other tumor entities. In breast cancer patients, the association with nodal status, as well as significant impact upon overall survival, highlights the clinical relevance of *SATB1*. Small molecules targeting *SATB1* may thus be a promising approach in treating breast cancer patients.

## Figures and Tables

**Table I:** Haplotype and genotype distribution in patients and controls.

A, Genotypes			

Genotype	Controls, n=121	Patients, n=241	P-value
-3600T>C			0.813
TT	48	88	
TC	57	117	
CC	16	36	
C frequency	0.37	0.39	
-3363A>G			0.187
AA	63	133	
AG	51	83	
GG	7	25	
G frequency	0.27	0.28	
-2984C>T			0.849
CC	80	165	
CT	38	69	
TT	3	7	
T frequency	0.18	0.17	

B, Haplotype frequency in cohort			

Haplotype	Controls	Patients	P-value

-3600 T/-3363 A/-2984 C	0.409	0.359	0.272
-3600 C/-3363 G/-2984 C	0.227	0.199	0.470
-3600 T/-3363 A/-2984 T	0.182	0.195	0.117
-3600 C/-3363 A/-2984 C	0.141	0.166	0.668
-3600 T/-3363 G/-2984 C	0.042	0.076	0.157

**Table II:** Linkage of *SATB1* polymorphisms in healthy controls.

	-3363A>G	-2984C>T
-3600T>C		
D′	0.744	1.000
r^2^	0.149	0.129
-2984C>T		
D′	1.000	
r^2^	0.744	

**Table III:** Clinical data and haplotype distribution.

		*SATB1*-3600T/-3363A/-2984C haplotype			
				
	All, n=241	Homozygous, n=30	Heterozygous, n=158	Other haplotypes, n=53	P-value
Median age at first diagnosis, years	56.3	55.9	56.1	56.9	0.877
Histopathological tumor type					0.532
Ductal	131 (54.4)	16 (53.3)	84 (53.2)	31 (58.5)	
Lobular	47 (19.5)	3 (10.0)	32 (20.3)	12 (22.6)	
Mixed lobular/ductal	38 (15.8)	6 (20.0)	27 (17.1)	5 (9.4)	
Other	25 (10.4)	5 (16.7)	15 (9.5)	5 (9.4)	
Median tumor size, mm	24.9	19.0	26.3	24.5	0.073
TNM status					
T					0.511
pT1	118 (49.4)	18 (60.0)	73 (46.8)	27 (50.9)	
pT2	94 (39.3)	11 (36.7)	61 (39.1)	22 (41.5)	
pT3	15 (6.3)	0	12 (7.7)	3 (5.7)	
pT4	12 (5.0)	1 (3.3)	10 (6.4)	1 (1.9)	
N					0.049
pN0	127 (52.7)	21 (70.0)	75 (47.5)	31 (58.5)	
pN+	114 (47.3)	9 (30.0)	83 (52.5)	22 (41.5)	
M					0.668
pM0	236 (97.9)	30 (100)	154 (97.5)	52 (98.1)	
pM1	5 (2.1)	0	4 (2.5)	1 (1.9)	
Grading					0.216
1	87 (37.3)	15 (51.7)	54 (35.3)	18 (35.3)	
2	93 (39.9)	10 (34.5)	66 (43.1)	17 (33.3)	
3	53 (22.7)	4 (13.8)	33 (21.6)	16 (31.4)	
Estrogen receptor status					0.839
Positive	132 (66.3)	14 (60.9)	88 (67.2)	15 (33.3)	
Negative	67 (33.7)	9 (39.1)	43 (32.8)	30 (66.7)	
Her2/neu status					0.493
Overexpression	26 (13.3)	3 (12.0)	19 (15.3)	4 (8.5)	
No overexpression	170 (86.7)	22 (88.0)	105 (84.7)	43 (91.5)	
Treatment					
Surgical					0.201
Breast-conserving	55 (22.8)	4 (13.3)	35 (22.2)	16 (30.2)	
Mastectomy	186 (77.2)	26 (86.7)	123 (77.8)	37 (70.8)	
Adjuvant					0.065
No medication	126 (52.3)	19 (63.3)	74 (46.8)	33 (62.3)	
Tamoxifen and/or CMF	115 (47.7)	11 (36.7)	84 (53.2)	20 (37.7)	
Median follow-up (months)	93.2	108.5	91.3	90.5	0.214

Values are presented as n (%), unless otherwise stated. CMF, cyclophosphamide, methotrexate and fluorouracil.
